# High-Density Linkage Map and Mapping for Sex and Growth-Related Traits of Largemouth Bass (*Micropterus salmoides*)

**DOI:** 10.3389/fgene.2019.00960

**Published:** 2019-10-10

**Authors:** Chuanju Dong, Peng Jiang, Jiangfan Zhang, Xuejun Li, Shengjie Li, Junjie Bai, Jiajia Fan, Peng Xu

**Affiliations:** ^1^Pearl River Fisheries Research Institute, CAFS, Guangzhou, China; ^2^College of Fisheries, Henan Normal University, Xinxiang, China; ^3^Key Laboratory of Tropical and Subtropical Fishery Resource Application and Cultivation, Ministry of Agriculture, Pearl River Fisheries Research Institute, CAFS, Guangzhou, China; ^4^State Key Laboratory of Marine Environmental Science, Xiamen University, Xiamen, China

**Keywords:** largemouth bass, linkage map, single-nucleotide polymorphism, sex determination, growth-related, quantitative trait loci

## Abstract

The largemouth bass is an important species, and its culture has risen sharply with the surge in fish aquaculture in China. Due to the lack of selective breeding technology for the largemouth bass, the growth rate and disease resistance are low, its sexual maturation is slow, and other serious problems are contributing to a sharp decline in the safety and quality of largemouth bass products in recent decades. Therefore, comprehensive breeding programs to improve the economic performance and promote the modern industrial development of largemouth bass must be considered a priority. Here, a total of 152 adult largemouth bass, including two parents and 150 progenies, were selected to produce the genetic mapping family. Then, a high-density linkage map was constructed based on restriction site–associated DNA sequencing using 6,917 single-nucleotide polymorphisms (SNPs) located in 24 linkage groups (LGs). The total genetic length of the linkage map was 1,261.96 cM, and the length of each LG varied from 24.72 cM for LG02 to 117.53 cM for LG16, with an average length of 52.58 cM and an average SNP number of 286. Thirteen significant quantitative trait loci (QTLs) for sex determination were located on LG04, LG05, LG08, LG12, LG15, LG21, and LG23. An informative QTL cluster that included six QTLs was detected on LG12. However, one notable QTL, which accounted for 71.48% of the total phenotypic variation, was located in the region of 1.85 cM on LG05. In addition, 32 identified QTLs were related to growth, including body weight, body length, body height, and head length. The QTLs for these growth-related traits are located in 13 LG regions and have little effect on phenotypic variation. This high-density genetic linkage map will enable the fine-mapping of economic traits and support the future genome assembly of the largemouth bass. Additionally, our study will be useful for future selective culture of largemouth bass and could potentially be used in molecular-assisted breeding of largemouth bass for aquaculture.

## Introduction

The largemouth bass, *Micropterus salmoides*, is a large carnivorous fish inhabiting freshwater rivers and lakes in North America (Bai et al., 2008). This species is now widely cultivated in China because of characteristics such as strong temperature resistance, adaptability to new environments, and attractiveness as a food source. In 1983, the largemouth bass was first introduced into mainland China. At present, it plays an indispensable role as a freshwater aquaculture species in China (Deng et al., 2011); more than 30 years after the establishment of largemouth bass farming, China has an annual output of approximately 500,000 tons. However, the lack of artificial selection and directional breeding in previous decades has resulted in a decline in the growth rate of pond-cultured fish (Dongmei et al., 2013), a shortened period of sexual maturity, and reduced disease resistance, which seriously affect the quality and safety of the fish products (Fogelson et al., 2016). In summary, a largemouth bass species program needs to be established to improve its economic traits and sustain the development of the largemouth bass industry.

One of the most common phenomena in biology is the strikingly different patterns of morphology, reproductive strategies, and behaviors exhibited by male and female individuals within a species (Songlin et al., 2014). Teleosts are the most widely distributed and diverse group of vertebrates. Unlike mammals and birds, which exhibit highly conserved master sex-determination genes that control a conserved genetic network responsible for gonad differentiation, teleosts exhibit a wide range of sex-determination patterns, which may be regulated by genetic and environmental factors and their interactions (Fowler and Buonaccorsi, 2016; Sun et al., 2017). From the perspective of evolution, how the transition between temperature and genetic determination takes place and why such a shift occurs remain a mystery, but they are a very interesting phenomenon (Shen and Wang, 2014). Generally, fish species show obvious sex determination. In some fish, significant differences in body size and growth rate exist between males and females, and these disparities can have a significant influence on their commercial value, such as the half-smooth tongue sole (*Cynoglossus semilaevis*) (Song et al., 2012), Japanese flounder (*Paralichthys olivaceus*) (Shao et al., 2015), halibut (Palaiokostas et al., 2013), tilapia (Andrey et al., 2006), and so on. The males of largemouth bass also have a better growth rate than the females. Therefore, identification of sex-associated markers can reveal the mechanisms underlying the genomic evolution and help determine sex for the generation of single-sex groups (Liao et al., 2009).

Linkage maps provide a framework for marker-assisted selection programs and have been widely used for quantitative trait locus (QTL) mapping of many important traits on chromosomes (Yue, 2014; Yu et al., 2016). Quantitative trait locus localization based on a high-density genetic linkage map can play an important role in promoting marker-assisted selection and breeding of aquaculture varieties (Liu and Cordes, 2004; Wang et al., 2006). In the process of constructing a high-density genetic linkage map (Salem et al., 2018), we gave priority to single-nucleotide polymorphisms (SNPs) because of their stability and abundance as genetic markers. The advent of a new generation of sequencing, including restriction site–related DNA sequencing (RAD-Seq), has revolutionized the genomic approach and allowed the discovery of thousands of SNPs throughout the genome (Davey et al., 2011; Houston et al., 2012). Using the methods mentioned above, QTL maps have been drawn to identify genetic structures associated with complex quantitative traits in many fish species (Palaiokostas et al., 2013; Sun et al., 2015), such as tilapia (Andrey et al., 2006; Shirak et al., 2006), common carp (Chen et al., 2018), kelp (Zhang et al., 2015), rainbow trout (Wang et al., 2011), halibut (Palaiokostas et al., 2013), Atlantic salmon (Fotherby et al., 2007), and other organisms (Kijas et al., 2018). These applications indicate that the RAD-Seq method is suitable for constructing high-density genetic linkage maps and QTL mapping (Palaiokostas et al., 2018).

When constructed successfully by RAD-Seq, a genetic map for a nonmodel species provides high efficiency at a low cost (Brown et al., 2016). Thus, the main purpose of this study is to construct the first high-density linkage map of largemouth bass as well as to fine-mapping for sex determination and growth in this species using RAD-Seq technology (Feng et al., 2018). Our study will contribute to the identification of potential genes for economic traits in largemouth bass; these genes can be used for sex determination and selective breeding. Our results will also support a new genome sequence assembly and comparative genome research in largemouth bass in the future.

## Materials and Methods

### Family Samples and DNA Isolation

The parents were collected from Foshan Nanhai Jieda Feed Co., Ltd., China. The largemouth bass F1 full-sib family was produced from the parental fish. Artificial injection of oxytocin was used to promote egg laying, and the collected fertilized eggs were placed in indoor incubators for incubation. The hatching water temperature was about 23°C, and the hatchlings were transferred to a cement pond with an area of 12 m^2^ for feeding. Small zooplankton and fairy shrimp were fed as an open bait, and then red worms were fed. When the largemouth bass was 3 months old, all the fish were put into one 667-m^2^ breeding pond. At the age of 9 months, 150 experimental fish were randomly collected from the progeny of the family for measurement of body weight (BW), body length (BL), body height (BH), and head length (HL) with an average BW of 313 g and BL of 23.9 cm. Then, anatomical method was carried out to determine the gender of each sample. At the same time, fin clips from the two parents and the muscle tissues of their progeny were preserved in absolute ethanol and stored in a −20°C freezer.

### RAD Library Construction and Sequencing

To construct a RAD library, genomic DNA from 152 samples (150 offspring and two parents) was extracted according to a previously published protocol (Baird et al., 2008), and its quality was evaluated with a Qubit Fluorimeter (Invitrogen, USA) and 0.6% agarose gel electrophoresis. The enzyme reaction system (30 μl) was incubated at 65°C for 10 min and contained 1 μg genomic DNA and 15 U of PstI (15 U/μl, restriction enzyme cut site 5′ CTGCA 3′) (Thermo Scientific, Waltham, MA, USA). Barcode adapters were designed with sample-specific nucleotide codes according to the standard Illumina adapter design flow (Illumina, San Diego, CA, USA). Afterward, the unique barcode adapters (10 μmol) for each DNA sample were added to the reaction system. Six pools containing all the DNA samples were assembled. Fragments with a size range from 250 to 450 bp were chosen to build the libraries. The six libraries were independently sequenced on different lanes of an Illumina HiSeq 4000 (Illumina) with 150-bp paired-end reads.

### SNP Calling and Genotyping

The Illumina sequencing short reads were filtered using the SOAPnuke package (http://soap.genomics.org.cn/) to remove reads containing more than 5% undetermined nucleotides and extremely low-quality bases. Unconstrained reads without sample-specific barcodes and enzyme motifs were discarded as well. Then, the high-quality reads were used for the subsequent analyses. The STACKS (version 0.99998) pipeline was used to assemble the loci, *de novo*, from the sequencing data for SNP calling (Rochette and Catchen, 2017). USTACKS, CSTACKS, SSTACKS, and GENOTYPE programs were utilized to create libraries of loci. A maximum of three mismatches was allowed between samples’ stacks when producing the catalog. A minimum depth of two sequencing reads was allowed for Stacks assembling. The modules Stacks and Genotypes were operated mapping each progeny data to the catalog for SNP calling and genotyping, respectively. The detailed parameters used were as follows: USTACKS: -i 1 -t gzfastq -r -d -m 2 -M 2 -p 15. CSTACKS: -b 1 -n 3 -p 15. SSTACKS: -b 1 –p 15. GENOTYPE: -b 1 -r 1 -t cp -o. Loci lost in more than 30% of the offspring were deleted from the subsequent analysis.

### High-Density Genetic Map Construction

Markers were initially grouped in JoinMap v4.1 using the “independence LOD” parameter setting as “Start = 2, End = 20, Step = 1” (Uchino et al., 2016). After this initial grouping to individual linkage groups (LGs), markers were divided into 24 groups. To reduce the needs for computing resources, we used Lep-MAP2 to calculate the genetic distance of each LG. The “Filtering” module was used to filter SNP markers by comparing the progenies genotype distribution and the expected Mendelian proportions (segregation distortion test), with “MAFLimit” set at 0.05 (consistent with filtering described above) and “dataTolerance” set at 0.001 to remove markers exhibiting significant segregation distortion. The “SeparateChromosomes” module was used to reassign markers into LGs. The “OrderMarkers” module with Kosambi mapping function was used to calculate the SNP markers order and genetic distance. Utilizing parallelized computing, this step was repeated several times to assess consistency of marker order between replicates. Meanwhile, error rates were calculated.

### QTL Fine-Mapping

QTL analyses were performed with WinQTLCart version 2.5 by using the composite interval mapping (CIM) model analysis. The LOD profiles were generated at 1-cM steps along each LG to identify the positive QTL position. Thresholds for LOD scores were determined by implementing a method of 500 permutation iterations with the *P* value less than 0.05. Thus, the linkage genome-wide LOD threshold was determined to 3.0. Quantitative trait locus that exceeded the LG-wide LOD threshold with *P* < 0.05 was recognized as suggestive QTL.

## Results

### Sequencing and Genotyping

After the low-quality reads had been filtered out, 354 Gigabytes (Gb) of clean data were obtained from the two parents and their 150 progeny (**Supplemental Table 1**). The average clean data of each progeny accounted for 2.5 Gb. After using the Stacks pipeline, we identified 60,577 SNP markers (28,040 maternal heterozygous, 25,125 paternal heterozygous, and 7,412 heterozygous in both parents; **Supplemental Table 2**) across all the progeny; of these markers, 18,480 SNP markers were present in more than 80% of the progeny. These refined SNP markers were used to construct the linkage map.

### Linkage Map Construction

**Figure 1:**
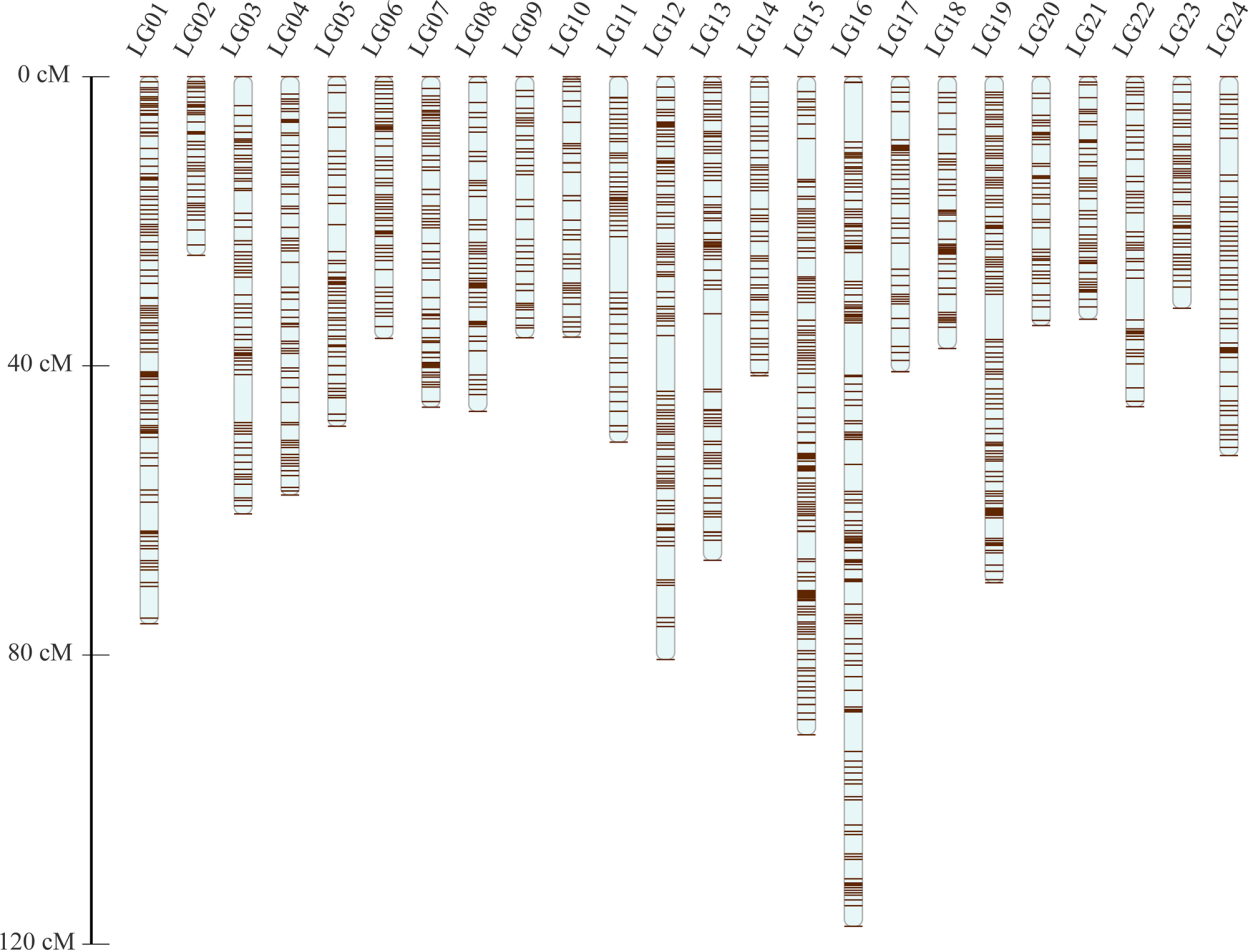
Genetic lengths and SNP distribution of 24 linkage groups of largemouth bass.

After the distorted segregation markers had been eliminated, 7,015 markers were improved and successfully assigned to 24 LGs, which was consistent with the reported karyotype analysis of largemouth bass. After correction with the Lep-MAP2 program, 98 SNPs were deleted. Finally, we generated the largemouth bass high-density genetic map with 6,917 SNPs and 24 LGs (**Figure 1** and **Table 1**, **Supplemental Table 3**). The linkage map of the total genomic length was 1,261.96 cM, with the genetic length of each LG varying from 24.72 cM for LG2 to 117.53 cM for LG16, with an average length of 52.58 cM and an average SNP number of 286.

**Table 1:** Summary statistics of the linkage map of largemouth bass.

LG	No. of SNPs	Distance (cM)
LG01	484	75.67
LG02	224	24.72
LG03	324	60.47
LG04	245	57.87
LG05	195	48.35
LG06	323	36.21
LG07	355	45.72
LG08	226	46.30
LG09	116	36.12
LG10	219	36.02
LG11	347	50.55
LG12	428	80.63
LG13	241	66.90
LG14	230	41.36
LG15	515	91.03
LG16	507	117.53
LG17	180	40.83
LG18	159	37.60
LG19	328	70.00
LG20	153	34.42
LG21	281	33.55
LG22	304	45.66
LG23	228	32.04
LG24 Total Average	245 6,857 286	52.41 1,261.96 52.58

### Mapping of Sex Determination

The high-density, high-resolution genetic linkage map consisted of 6,857 SNPs, approximately 100-fold more markers than the previous linkage map. This linkage map will be applied widely to mapping and positional cloning of important traits, thereby providing a new chromosome framework for the assembly and map integration of genome sequences.

**Figure 2:**
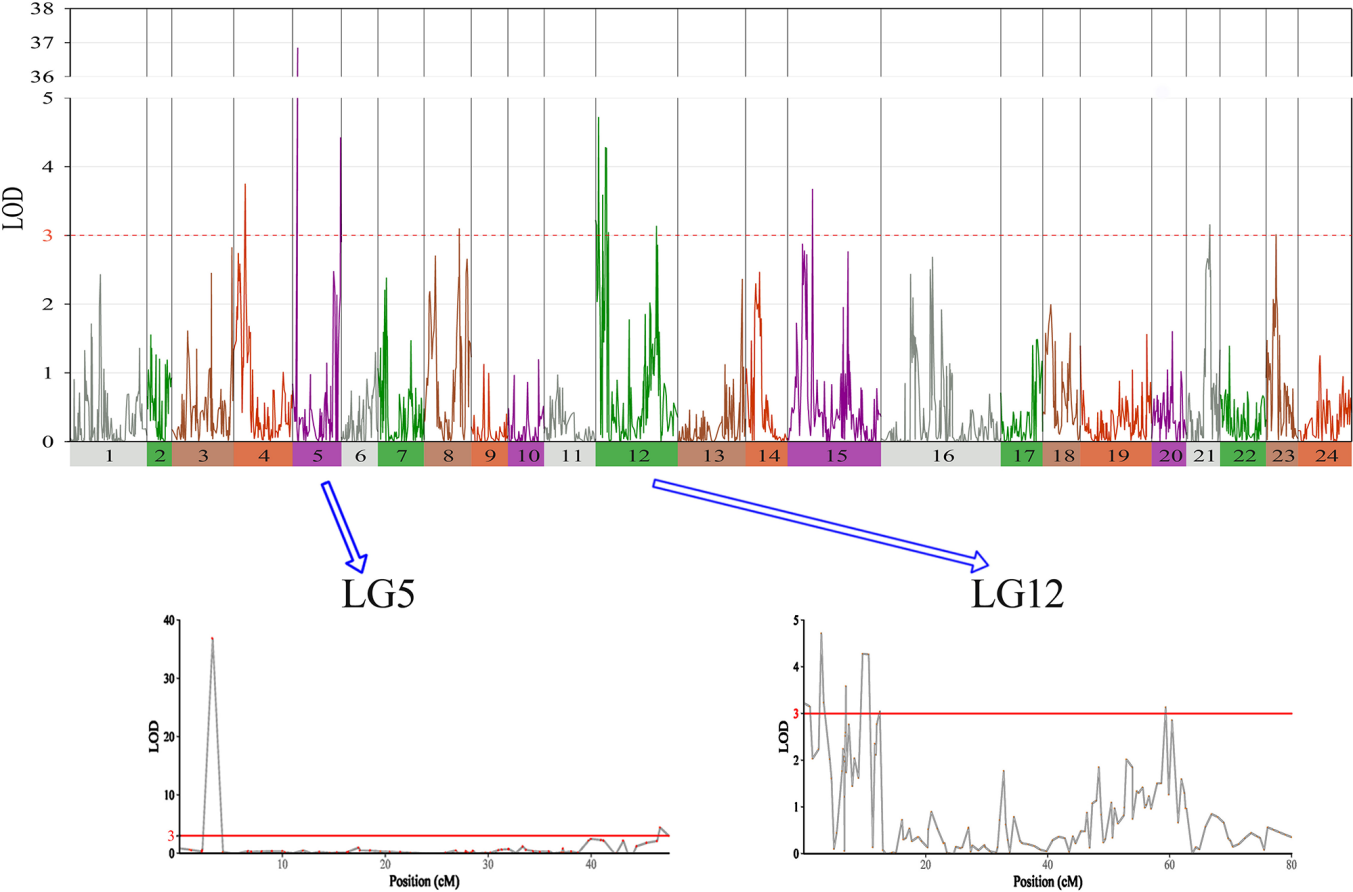
The sex determination in largemouth bass, where chromosome-wide LOD (3.0) threshold is denoted with a red dashed line.

In this study, we identified 13 significant QTLs, including 49 SNP loci for sex determination, which were distributed among seven LGs: LG04, LG05, LG08, LG12, LG15, LG21, and LG23. The most significant QTL had an LOD value of 36.87 and accounted for 71.48% of the phenotypic variation. It is located in the 1.85-cM region of LG05 and is supported by two markers clustered together on LG05. In addition, an adjacent QTL accounts for 11.51% of the total phenotypic variation; its LOD value is 3.75, and it contains four markers. Several QTLs (QTL5, QTL6, QTL7, QTL8, QTL9, and QTL10) were also clustered on LG12 and supported by 26 absolute markers (**Figure 2**, **Table 2**, **3**).

**Table 2:** Genomic regions associated with sex determination in largemouth bass.

QTL peak locus	LG	Position (cM)	LOD	Explained variation	No. of SNPs
QTL1-SD	LG04	10.71–11.54	3.75	11.51%	4
QTL2-SD	LG05	2.20–4.15	36.87	71.48%	2
QTL3-SD	LG05	46.53–47.53	4.42	7.97%	3
QTL4-SD	LG08	34.28–34.33	3.1	8.51%	1
QTL5-SD	LG12	0–1.07	3.15	14.13%	1
QTL6-SD	LG12	2.58–3.46	4.72	8.25%	14
QTL7-SD	LG12	6.87–6.93	3.59	9.91%	1
QTL8-SD	LG12	9.33–10.86	4.28	11.90%	7
QTL9-SD	LG12	12.40–12.49	3.04	8.80%	2
QTL10-SD	LG12	59.30–59.40	3.13	7.42%	1
QTL11-SD	LG15	24.02–24.39	3.67	0.65%	1
QTL12-SD	LG21	22.75–23.09	3.16	7.09%	6
QTL13-SD	LG23	9.70–9.72	3.01	8.85%	6

**Table 3:** Details of genotype for SNPs and the number of male and female fish of QTL2-SD in largemouth bass.

Marker ID	Genotype	No. of females	No. of males	Ratio of female to male
768973	CT	68	0	1.08
	TT	0	63	
410100	AG	71	0	1.11
	AA	0	64	

Among these, the highest LOD value for sexual determination is 4.72 for QTL6, which is located at 2.58 to 3.46 cM and accounts for 8.25% of the phenotypic variation. In addition, QTL5 is located at 0 to 1.07 cM and accounts for 14.13% of the phenotypic variation and has an LOD value of 3.15. The other QTLs on LG12 for sexual determination were found at 6.87 to 6.93, 9.33 to 10.86, 12.40 to 12.49, and 59.30 to 59.40 cM; these QTLs had LOD values of 3.04 to 4.28 and accounted for 8.80% to 11.90% of the phenotypic variation.

### Growth-Related Locus Detection

**Figure 3:**
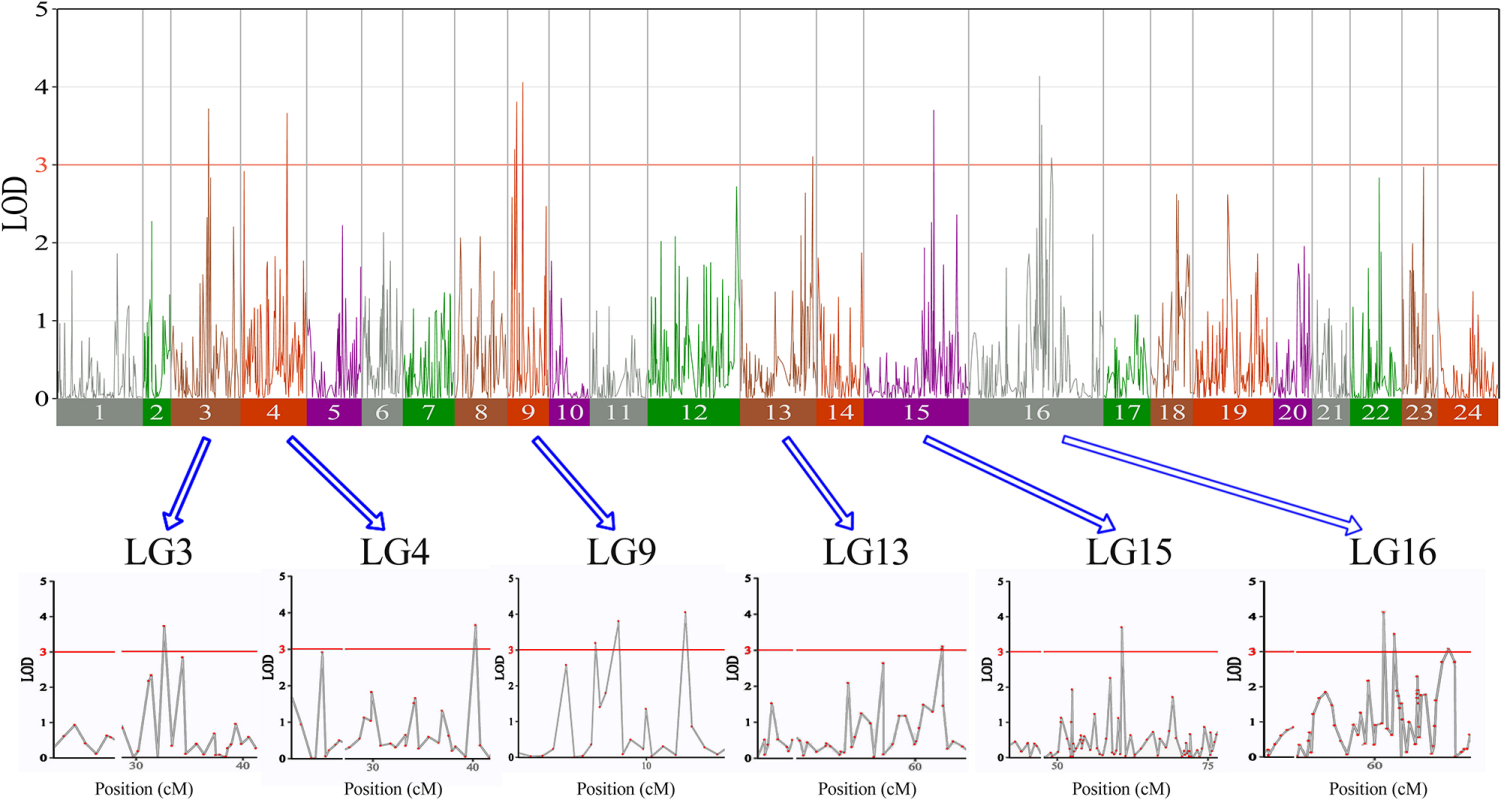
The body weight in largemouth bass, where chromosome-wide LOD (3.0) threshold is denoted with a red dashed line.

**Figure 4:**
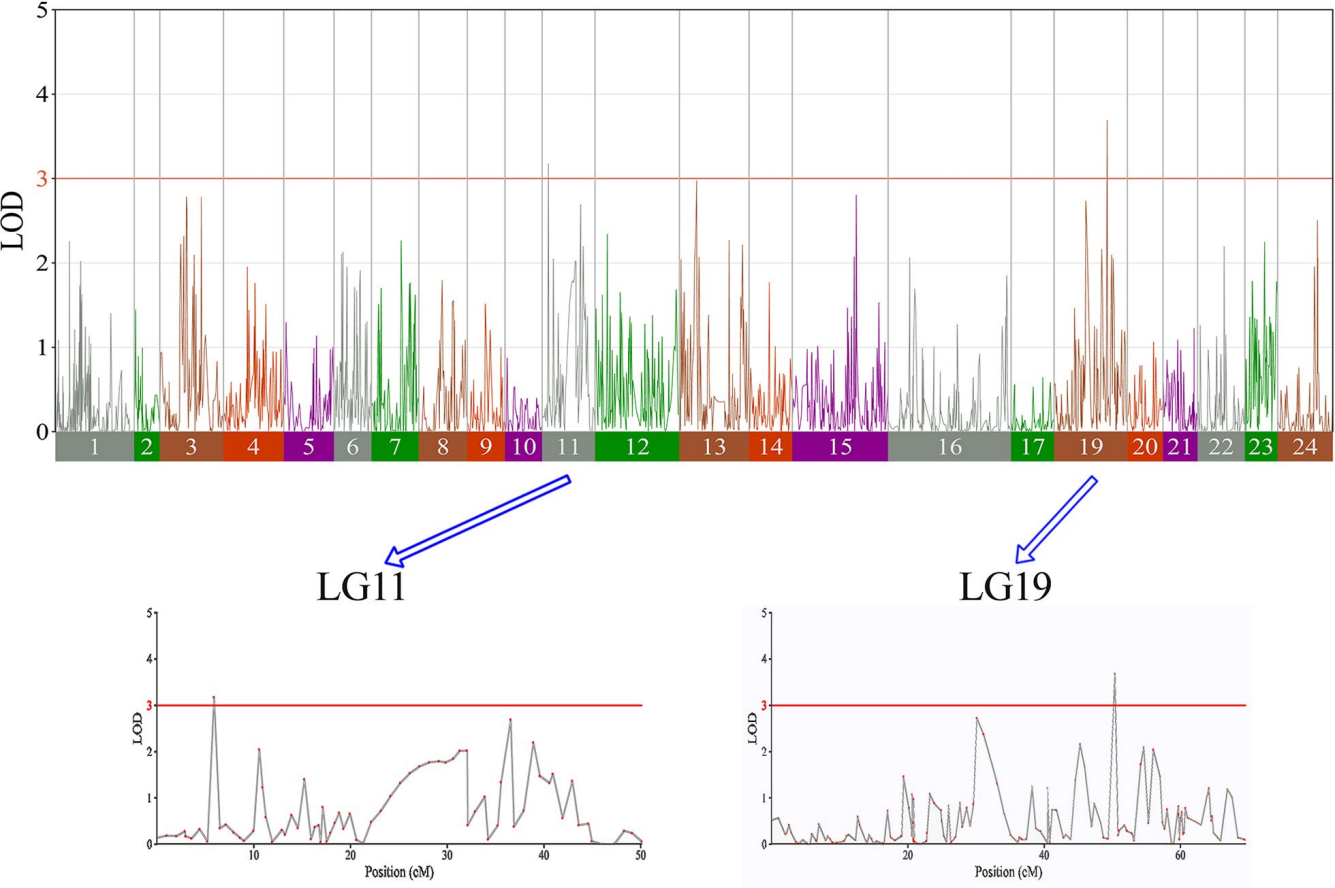
The body length in largemouth bass, where chromosome-wide LOD (3.0) threshold is denoted with a red dashed line.

**Figure 5:**
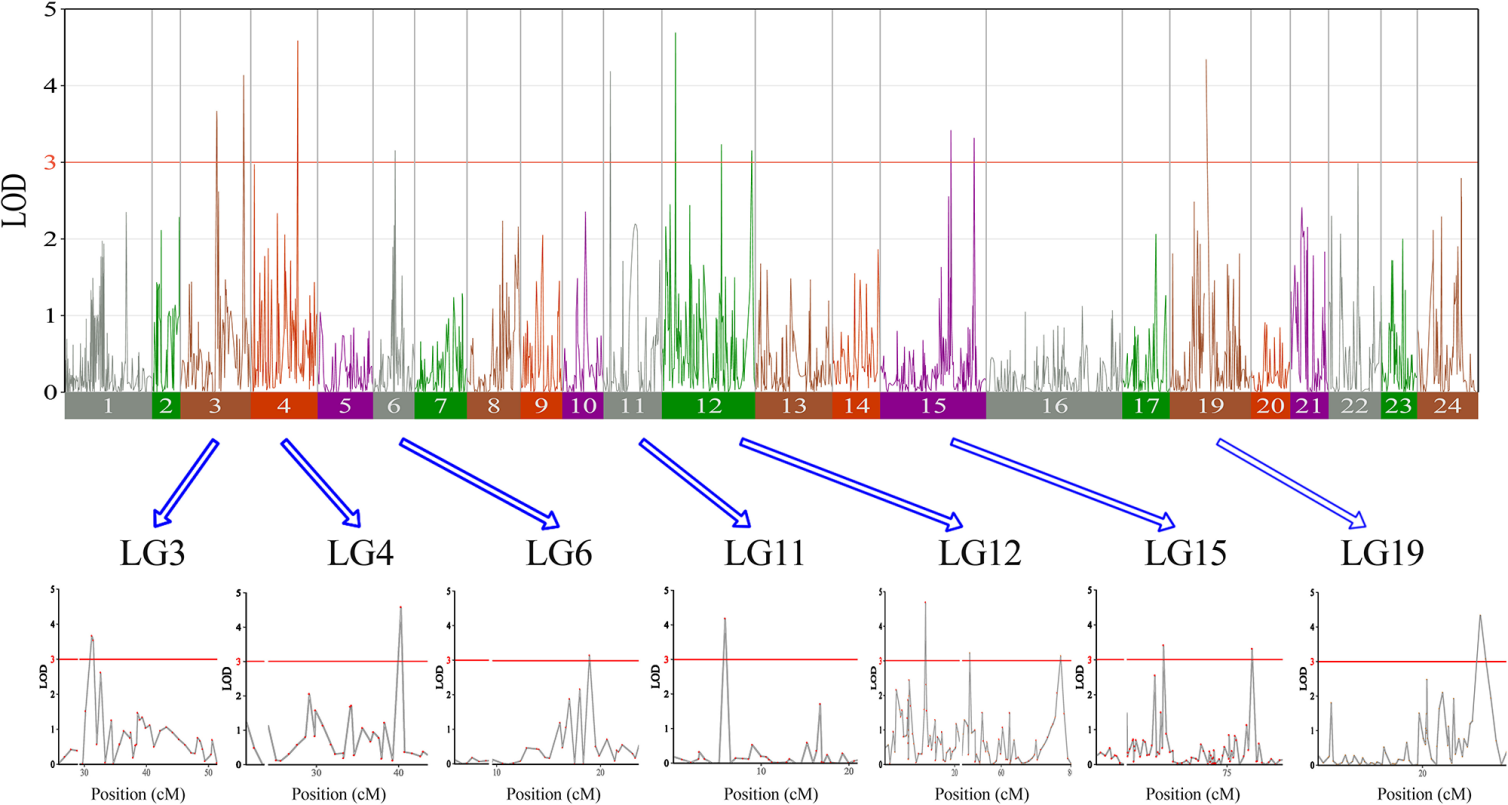
The head length in largemouth bass, where chromosome-wide LOD (3.0) threshold is denoted with a red dashed line.

**Figure 6:**
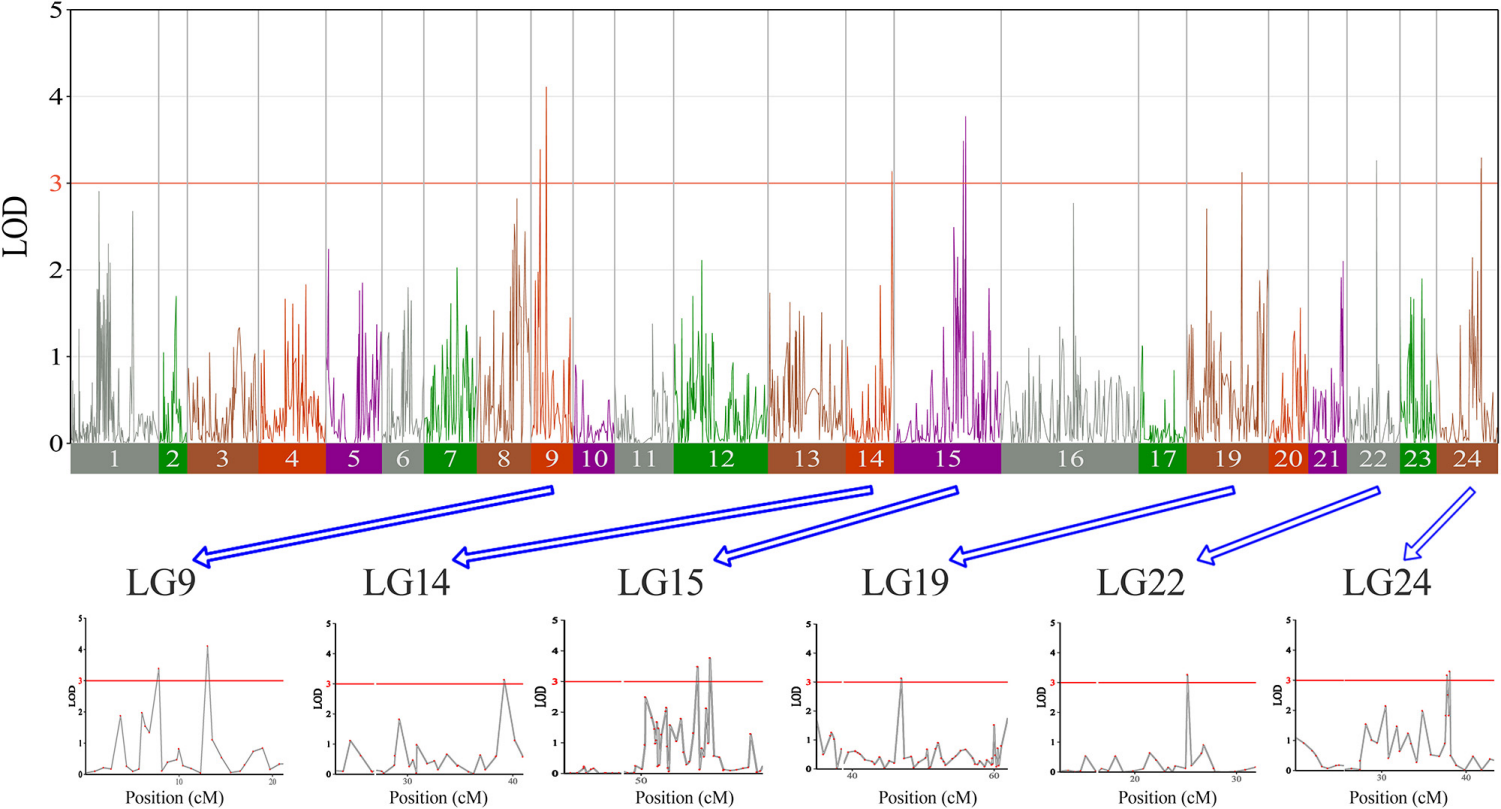
The body height in largemouth bass, where chromosome-wide LOD (3.0) threshold is denoted with a red dashed line.

In this study, we investigated BW, BL, HL, and BH as growth-related traits and identified growth-related QTLs on 13 LGs. These 32 QTL regions related to growth-related traits were found to harbor 202 significant SNPs, including 36 SNPs for BW, 53 SNPs for BL, 86 SNPs for HL, and 27 SNPs for BH. The large distribution and low phenotypic variation of these QTLs indicate that they are controlled by complex genetic regulation during growth. Ten QTLs for BW were identified, and they explained between 6.11% and 14.99% of the total phenotypic variance. The most prominent region had the highest LOD value at 4.14; it was located at 61.3 to 61.7 cM of LG16 and accounted for a notable 11.55% of phenotypic variation (**Figure 3** and **Table 4**). The two QTLs for BL were located on two different LG regions and explained between 8.56% and 10.56% of the phenotypic variation (**Figure 4** and **Table 5**); the most prominent was located at 50.0 to 50.6 cM of LG19 with an LOD value of 3.69. For HL, 11 QTLs were found, and these accounted for 9.57% to 13.97% of the total phenotypic variance (**Figure 5** and **Table 6**). For BH, nine QTLs were found on six LGs, and these accounted for 8.89% to 13.03% of the total phenotypic variance (**Figure 6** and **Table 7**). Although the QTL profiles of the four growth-related traits showed significant differences, a few overlaps were present. For instance, BW2 shared almost the same interval as HL3, BW4 shared almost the same interval as BH1, and the BW5 region was included in BH2. Additionally, the BW7, the smallest region, ranged from 60.7 to 61.0 cM of LG15, exactly the same region as BH5 and similar to HL10.

**Table 4:** Genomic regions associated with Body weight in largemouth bass.

QTL peak locus	LG	Position (cM)	LOD	Explained variation	No. of SNPs
QTL1-BW	LG03	32.4–32.9	3.72	10.79%	12
QTL2-BW	LG04	40.0–40.5	3.66	11.59%	3
QTL3-BW	LG09	6.0–6.2	3.2	10.63%	1
QTL4-BW	LG09	7.2–8.0	3.81	14.99%	3
QTL5-BW	LG09	12.8–13.3	4.06	12%	3
QTL6-BW	LG13	62.6–63.0	3.04	8.41%	1
QTL7-BW	LG15	60.7–61.0	3.7	6.11%	12
QTL8-BW	LG16	70.7–72.9	3.09	14.65%	3
QTL9-BW	LG16	63.2–63.5	3.51	10.37%	1
QTL10-BW	LG16	61.3–61.7	4.14	11.55%	1

**Table 5:** Genomic regions associated with body length in largemouth bass.

QTL peak locus	LG	Position (cM)	LOD	Explained variation	No. of SNPs
QTL1-BL	LG11	5.7–6.0	3.17	8.56%	53
QTL2-BL	LG19	50.0–50.6	3.69	10.56%	8

**Table 6:** Genomic regions associated with head length in largemouth bass.

QTL peak locus	LG	Position (cM)	LOD	Explained variation	No. of SNPs
QTL1-HL	LG03	30.6–31.6	3.67	11.26%	1
QTL2-HL	LG03	53.9–54.8	4.13	10.13%	10
QTL3-HL	LG04	39.8–40.6	4.58	13.97%	3
QTL4-HL	LG06	18.8–19.1	3.15	13.38%	1
QTL5-HL	LG11	5.6–6.2	4.18	10.01%	53
QTL6-HL	LG12	76.6–77.5	3.15	17.57%	1
QTL7-HL	LG12	50.8–51.2	3.23	16.67%	2
QTL8-HL	LG12	11.4–11.9	4.69	16.6%	2
QTL9-HL	LG15	80.3–80.9	3.32	10.26%	12
QTL10-HL	LG15	60.7–61.0	3.41	9.57%	2
QTL11-HL	LG19	29.8–32.6	5.26	13.56%	1

**Table 7:** Genomic regions associated with body height in largemouth bass.

QTL peak locus	LG	Position (cM)	LOD	Explained variation	No. of SNPs
QTL1-BH	LG09	7.4–7.9	3.39	13.03%	3
QTL2-BH	LG09	12.7–13.3	4.11	12.19%	3
QTL3-BH	LG14	39.0–39.5	3.13	12.84%	1
QTL4-BH	LG15	58.5–59.0	3.48	13.19%	3
QTL5-BH	LG15	60.7–61.1	3.77	11.57%	12
QTL6-BH	LG19	46.7–47.1	3.12	22.52%	6
QTL7-BH	LG22	25.1–25.3	3.26	8.89%	6
QTL8-BH	LG24	37.7–37.9	3.17	9.67%	1
QTL9-BH	LG24	38.0–38.2	3.29	9.41%	1

## Discussion

Comparing with other genetic markers, SNP markers are the most abundant type of marker in the genome with high polymorphism. However, in a nonmodel aquaculture species with a relatively large mapping population, developing a sufficient number of SNPs and performing cost-effective genotyping are difficult (Palaiokostas et al., 2013; Zhao et al., 2017). With advances of the NGS technologies, various sequence-based SNP genotyping technologies have been developed providing rapid and cost-effective high-throughput SNP genotyping platforms for linkage mapping. In many species, such as the common carp (Jian et al., 2014), turbot (Bouza et al., 2012), and rice (Deschamps et al., 2010), the detection of SNPs has increased significantly and contributes to a better understanding of complex traits, genetic breeding, and genomic selection. RAD-Seq is a useful method for the discovery of SNP markers that is being extensively used in many species (Baird et al., 2008; Davey et al., 2013). If a reference genome is unavailable, the *de novo* method is one of the best approaches to use with the available analytical tools (Baxter et al., 2011; Catchen et al., 2011). Therefore, most researchers can perform RAD-Seq in dozens of individuals from an F1 hybrid population as well as their parents (Yue, 2014), such as catfish (Yun et al., 2015), common carp (Xiaofeng et al., 2013), Asian sea bass (Le et al., 2015), Atlantic salmon (Gonen et al., 2014), rainbow trout (Nichols et al., 2015), Japanese freshwater fish (Shao et al., 2015), and many others. As one of the important applications of genetic mapping, QTL mapping provides a bridge from structural genome research to functional genome research and has important applications in production. By providing a framework for QTL mapping, high-quality genetic linkage maps can facilitate genetic breeding of economically important aquaculture varieties (Chen et al., 2018). In Japanese ﬂounder, lymphocystis disease-resistance traits have been localized with a high-quality genetic linkage map and applied to marker-assisted breeding (Fuji et al., 2006). In salmon (Reid et al., 2005) and rainbow trout (Sundin et al., 2005), growth-related traits have been located and studied with a genetic map. A number of linkage maps of common carp have been constructed, and immediately afterward, different QTLs for body shape, swimming ability, meat quality, and so on were successfully mapped (Zhang et al., 2011; Laghari et al., 2014), etc.

In this study, with the help of RAD-seq method, we were able to take advantage of our novel genotyping array, which contained 6,917 SNPs and provided new insights into sex-related traits. As the first high-density genetic map of largemouth bass, this map provides an essential tool for QTL fine-mapping of sex determination. One major QTL (LG05) associated with sex determination was supported by two markers, which clustered in a narrow linkage span (2.20–4.15 cM) of LG05 and accounted for 71.48% of the total phenotypic variation, suggesting that this is one of the most important QTLs for sex determination in largemouth bass (**Figure 3**). Moreover, other QTLs (QTL5, QTL6, QTL7, QTL8, QTL9, and QTL10) were found to be clustered on LG12 and were supported by an aggregation of 26 markers (**Figure 3**). From the details of genotype for SNPs and the number of male and female fish of QTL2-SD in largemouth bass, we found that these two markers suggested that this species may have monogenic sex determination of ZW (female) and ZZ (male) kind. Also, other regions are most likely to be “micro effect function,” which just makes interference caused by other factors. Normally, heterogametic sex chromosomes have evolved independently in various lineages of vertebrates, with one of the chromosomes harboring master sex-determining genes or major loci. Such phenomena are sometimes found in fish sex determination studies. For example, Takashi Kamiya found that an SNP (C/G) in the Amhr2 gene is the only polymorphism associated with phenotypic sex by linkage and association mapping of the sex-determining locus in tiger pufferfish (*Takifugu rubripes*). They also discovered that two other species of *Takifugu* are conserved with the association of the Amhr2 SNP with phenotypic sex. Such undifferentiated sex chromosomes may be more common in vertebrates than previously thought (Takashi et al., 2012). We also found similar phenomena in the mandarin fish; one major QTL (LG23) related to sex determination was supported by five markers that were found to cluster at a narrow linkage span (60.27–68.71 cM) of LG23, contributing to 37.1% to 53.3% of the phenotypic variation, indicating that this was the sex determination (Sun et al., 2017). In Atlantic halibut, four significant sex-determination markers were located in the 3.2-cM region of LG13, in which 78% to 89% of the markers were related to sex (Palaiokostas et al., 2013). In addition, two important QTLs that influence sex determination in the Gilthead sea bream are located on LG21 (Dimitrios Loukovitis, 2012). In contrast, for some teleosts, sex-related QTLs are thought to be distributed on different LGs. In sea bass, hypothesized sex-determination QTLs existed on LG6, LG11, and LG18-21, accounting for 16.19% to 21.28% of the variation in traits (Palaiokostas et al., 2015). Seven sex-related loci were mapped in LG1f, LG14f, and LG1m by CIM in the half-smooth tongue sole, and they accounted for 12.5% to 25.2% of the trait variation (Song et al., 2012). Also, in tilapia and turbot, sex-determining QTLs were identified in association with different chromosomes (Martínez et al., 2009; Eshel et al., 2012). The above research results indicate that single or multiple LGs or chromosomes are involved in sex determination in different species, providing support for monogenic or polygenic sex determination. Therefore, the mechanisms of sex determination of teleosts are extremely complicated and remain to be further elucidated. However, in this study, the high LOD values and phenotypic variations in the major sex-related QTLs in this study suggest that a single LG may be involved in sex determination in largemouth bass and that one sex-determining gene may be located in this LG.

For years, researchers have tried to unravel the genetic basis of growth-related measurements, not only because of their high biological value but also owing to their economic importance. The ultimate goal is to develop molecular markers that can predict the phenotypes of edible fish and eventually apply these markers to marker-assisted breeding. However, for now, we still know little about the relationships between the loci of effect or genes and the growth-related traits in most of teleosts (Chen et al., 2018). In this study, 32 QTLs in total were found to be associated with four growth traits. However, compared to the high contributions of the major sex-related QTL, the contributions of each growth-related QTLs were quite low (6.11%–14.99%). This result was not surprising, because multiple QTLs contribute to these complex traits. The QTLs associated with four growth traits (BW, BL, BH, and HL) in the largemouth bass were analyzed. Multiple QTLs for a single growth trait may be distributed on different LGs, and different QTLs supported by different trait markers can be detected on multiple LGs. For instance, significant QTLs for the BW were detected on seven and five different LGs in rainbow trout and Asian sea bass, respectively. In turbot, 11 QTLs for BL in the second growth stage were detected on eight different LGs, indicating that the quantitative variation of fish growth traits was controlled by multiple separate major QTLs.

Here, we found that QTLs in certain chromosomal regions were correlated with multiple growth traits in largemouth bass. Quantitative trait loci controlling the BW and HL were present in the same regions of LG04 and LG15. The markers on LG04 were identical, and both significantly correlated with BW and HL, as well as the markers on LG15; this result demonstrated the similarity of these two traits, in agreement with the result of the phenotypic comparison, which showed that higher HL was correlated with higher BW. Similar studies have been reported in the Songpu mirror carp, Yellow River carp, and related hybrids, with few molecular markers (Yunjing et al., 2014). HL has been reported to be important in determining the BW of rounded fish (Neto et al., 2012), and HL has a greater direct impact on BW than on BL in large yellow croaker (Liu, 2008).

Additionally, the QTLs for BW and BH were identified in almost the same region of LG09, as well as LG15, indicating that a strong correlation exists between BW and BH, and BH may be an indirect indicator of BW in largemouth bass. In addition to two growth traits associated in some chromosomal regions of a single QTL, the QTL BW7, which was mapped to the smallest region from 60.7 to 61.0 cM of LG15, shared exactly the same region with BH5 and had a similar interval to HL10; thus, this location was found to be associated with three growth traits. Markers in these three QTLs coincided and were significantly correlated with BW, BH, and HL. This phenomenon, where a single QTL is associated with two or more growth traits in some chromosome regions, is common. In the mandarin fish, the marker R1_32378 on LG10 was significantly correlated with both BW and BH (Sun et al., 2017). In the golden pompano, all of the remaining BH QTLs explained 16.6% to 21.3% of the phenotypic variation, thus revealing a QTL common to BW, BH, and BL (Zhang et al., 2017). In the Asian sea bass, Lca371 on LG02 was shown to be associated with BW, BL, and total length (Wang et al., 2011). In the turbot, the SNP locus SM_343 on LG1 displayed a significant QTL effect during the first and second growth periods and was responsible for two or more traits with an LOD greater than 5.0 (Wang et al., 2015). In the common carp, SNP0626 on LG19 was significantly related to both BW and BL (Laghari et al., 2014). Similar phenomena have been observed in some fish, suggesting that genes in that particular region have significant pleiotropic effects, controlling a series of related traits. Moreover, the identification of QTLs influencing many traits could enhance genetic progress and molecular marker–assisted breeding and imply that a genetic interaction underlies these traits in aquaculture (Niu et al., 2017).

## Conclusion

Herein, we performed high-throughput SNP genotyping on a mapping family of largemouth bass and constructed a high-density genetic linkage map. This map provides an important tool for QTL fine-mapping of economically important traits. We were able to take advantage of a high-throughput SNP genotyping array to provide new insights into sex- and growth-related traits and determine the number and position of QTLs for these traits, although candidate gene identification was difficult because of the absence of a reference genome. Therefore, further molecular cloning to examine the locations of the QTLs in this study or whole-genome sequencing and gene annotation of largemouth bass is the ideal solution for identifying the candidate genes, and these strategies should be employed in the future.

## Data Availability Statement

All raw sequencing data have been deposited in the NCBI Sequence Read Archive (SRA: PRJNA528165).

## Ethics Statement

This study was approved by the Animal Conservation and Utilization Committee of Fisheries College of Henan Normal University. All methods were carried out in accordance with the approved guidelines.

## Author Contributions

SL conceived the project and designed the scientific objectives. CD, JF, and PJ collected and prepared the fish samples. CD and JZ conducted bioinformatics analysis. CD and SL prepared the manuscript. JB, XL, and PX revised the manuscript. All authors have read and approved the final manuscript.

## Funding

This research was supported by the Special Scientific Research Funds for Central Non-profit Institutes, Chinese Academy of Fishery Sciences (2016HY-ZC0403); the Fishing Port Construction and Fishery Industry Development Project (No.A201601A12); National Natural Science Foundation of China (31801032, 31602149); Open project of National and Local Joint Engineering Laboratory for Freshwater Fish Breedin (KF-2016-03); Science and Technology Research of Henan province (172102210348, 182102210081); and by the PhD Foundation of Henan Normal University (qd16159).

## Conflict of Interest

The authors declare that the research was conducted in the absence of any commercial or financial relationships that could be construed as a potential conflict of interest.

The handling editor declared a past co-authorship with one of the authors PX.

## References

[B1] Andrey S. Eyal S. Avner C. Howe A. E. Raisa D. Noam Z. (2006). Amh and Dmrta2 genes map to tilapia (*Oreochromis* spp.) linkage group 23 within quantitative trait locus regions for sex determination. Genetics 174 (3), 1573–1581. 10.1534/genetics.106.059030 16951079 PMC1667067

[B2] Bai J. Lutz-Carrillo D. J. Quan Y. Liang S. (2008). Taxonomic status and genetic diversity of cultured largemouth bass *Micropterus salmoides* in China. Aquaculture 278 (1-4), 0–30. 10.1016/j.aquaculture.2008.03.016

[B3] Baird N. A. Etter P. D. Atwood T. S. Currey M. C. Shiver A. L. Lewis Z. A. (2008). Rapid SNP discovery and genetic mapping using sequenced RAD markers. Plos One 3 (10), e3376. 10.1371/journal.pone.0003376 18852878 PMC2557064

[B4] Baxter S. W. Davey J. W. Spencer J. Shelton A. M. Heckel D. G. Jiggins C. D. (2011). Linkage mapping and comparative genomics using next-generation RAD sequencing of a non-model organism. Plos One 6 (4), e19315. 10.1371/journal.pone.0019315 21541297 PMC3082572

[B5] Bouza C. Hermida M. Pardo B. G. Vera M. Fernández C. Herrán R. D. L. (2012). An expressed sequence tag (EST)–enriched genetic map of turbot (*Scophthalmus maximus*): a useful framework for comparative genomics across model and farmed teleosts. BMC Genet. 13 (1), 54. 10.1186/1471-2156-13-54 22747677 PMC3464660

[B6] Brown J. K. Taggart J. B. Bekaert M. Wehner S. Palaiokostas C. Setiawan A. N. (2016). Mapping the sex determination locus in the hāpuku (*Polyprion oxygeneios*) using ddRAD sequencing. BMC Genomics 17 (1), 448. 10.1186/s12864-016-2773-4 27286864 PMC4902995

[B7] Catchen J. M. Amores A. Hohenlohe P. (2011). “Stacks: building and genotyping loci *de novo* from short-read sequences,” in G3 (Bethesda, Md.) 1 (3), 171–182. 10.1534/g3.111.000240 PMC3276136 22384329

[B8] Chen L. Peng W. Kong S. (2018). Genetic mapping of head size related traits in common carp (*Cyprinus carpio*). Front. Genet. 9, 38. 10.3389/fgene.2018.00448 30356829 PMC6190898

[B9] Davey J. W. Hohenlohe P. A. Etter P. D. (2011). “Genome-wide genetic marker discovery and genotyping using next-generation sequencing,” in Genetics. NATURE REVIEWS GENETICS. 12 (7), 499-510. 10.1038/nrg3012 21681211

[B10] Davey J. W. Timothée C. Pablo F. U. Cathlene E. Karim G. Blaxter M. L. (2013). Special features of RAD sequencing data: implications for genotyping. Mol. Ecol. 22 (11), 3151–3164. 10.1111/mec.12084 23110438 PMC3712469

[B11] Deng G. Shengjie L. I. Xie J. Bai J. Chen K. Dongmei M. A. (2011). Characterization of a ranavirus isolated from cultured largemouth bass (*Micropterus salmoides*) in China. Aquaculture 312 (1), 198–204. 10.1016/j.aquaculture.2010.12.032

[B12] Deschamps S. Rota M. L. Ratashak J. P. Biddle P. Thureen D. Farmer A. (2010). Rapid genome-wide single nucleotide polymorphism discovery in soybean and rice *via* deep resequencing of reduced representation libraries with the Illumina genome analyzer. Plant Genome 3 (1), 53–68. 10.3835/plantgenome2009.09.0026

[B13] Dongmei M. Guocheng D. Junjie B. Shengjie L. Lingyun Y. Yingchun Q. (2013). A strain of *Siniperca chuatsi rhabdovirus* causes high mortality among cultured largemouth bass in South China. J. Aquat. Anim. Health 25 (3), 197–204. 10.1080/08997659.2013.799613 23915177

[B14] Eshel O. Shirak A. Weller J. I. (2012). “Linkage and physical mapping of sex region on LG23 of Nile tilapia (*Oreochromis niloticus*),” in G3 (Bethesda, Md.). 2 (1), 35–42. 10.1534/g3.111.001545 PMC3276181 22384380

[B15] Feng X. Yu X. Fu B. Wang X. Liu H. Pang M. (2018). A high-resolution genetic linkage map and QTL fine mapping for growth-related traits and sex in the Yangtze River common carp (*Cyprinus carpio haematopterus*). BMC Genomics 19 (1), 230. 10.1186/s12864-018-4613-1 29609551 PMC5879560

[B16] Fogelson S. B. Petty B. D. Reichley S. R. Ware C. Bowser P. R. Crim M. J. (2016). Histologic and molecular characterization of *Edwardsiella piscicida* infection in largemouth bass (*Micropterus salmoides*). J. Vet. Diagn. Invest. Off. Publ. Am. Assoc. Vet Lab. Diagn. Inc. 28 (3), 1040638716637639. 10.1177/1040638716637639 26951328

[B17] Fotherby H. A. Moghadam H. K. Danzmann R. G. Ferguson M. M. (2007). Detection of quantitative trait loci for body weight, condition factor and age at sexual maturation in North American Atlantic salmon (*Salmo salar*) and comparative analysis with rainbow trout (*Oncorhynchus mykiss*) and Arctic charr (*Salvelinus alpinus*). Aquaculture 272 (6), S256–S257. 10.1016/j.aquaculture.2007.07.060 17308931

[B18] Fowler B. L. S. Buonaccorsi V. P. (2016). Genomic characterization of sex—identification markers in *Sebastes carnatus* and *Sebastes chrysomelas* rockfishes. Mol. Ecol. 25 (10), 2165–2175. 10.1111/mec.13594 26923740

[B19] Fuji K. Kobayashi K. Hasegawa O. Coimbra M. R. M. Sakamoto T. Okamoto N. (2006). Identification of a single major genetic locus controlling the resistance to lymphocystis disease in Japanese flounder (*Paralichthys olivaceus*). Aquaculture 254 (1), 203–210. 10.1016/j.aquaculture.2005.11.024

[B20] Gonen S. Lowe N. R. Cezard T. Gharbi K. Bishop S. C. Houston R. D. (2014). Linkage maps of the Atlantic salmon (*Salmo salar*) genome derived from RAD sequencing. BMC Genomics 15 (1), 166. 10.1186/1471-2164-15-166 24571138 PMC4028894

[B21] Houston R. D. Davey J. W. Bishop S. C. Lowe N. R. Mota-Velasco J. C. Hamilton A. (2012). Characterisation of QTL-linked and genome-wide restriction site-associated DNA (RAD) markers in farmed Atlantic salmon. BMC Genomics 13 (1), 244. 10.1186/1471-2164-13-244 22702806 PMC3520118

[B22] Jian X. Zhao Z. Zhang X. Zheng X. Li J. Jiang Y. (2014). Development and evaluation of the first high-throughput SNP array for common carp (*Cyprinus carpio*). BMC Genomics 15 (1), 307. 10.1186/1471-2164-15-307 24762296 PMC4234440

[B23] Kijas J. Mcwilliam S. Naval M. S. Kube P. King H. Evans B. (2018). Evolution of sex determination loci in Atlantic salmon. Sci. Rep. 8 (1), 5664. 10.1038/s41598-018-23984-1 29618750 PMC5884791

[B24] Laghari M. Y. Lashari P. Zhang X. Xu P. Xin B. Zhang Y. (2014). Mapping quantitative trait loci (QTL) for body weight, length and condition factor traits in backcross (BC1) family of common carp (*Cyprinus carpio* L.). Mol. Biol. Rep. 41 (2), 721–731. 10.1007/s11033-013-2911-x 24368591

[B25] Le W. Zi Y. W. Bai B. Shu Q. H. Chua E. Lee M. (2015). Construction of a high-density linkage map and fine mapping of QTL for growth in Asian seabass. Sci. Rep. 5 (5), 16358. 10.1038/srep16358 26553309 PMC4639833

[B26] Liao X. Ma H.Y. Xu G.B. Shao C.W. Tian Y.S. Ji X.S. (2009). Construction of a genetic linkage map and mapping of a female-specific DNA marker in half-smooth tongue sole *(Cynoglossus semilaevis)*. Mar. Biotechnol. 11 (6):699-709. 10.1007/s10126-009-9184-3 19214631

[B27] Liu X. D. (2008). The correlation and path analysis for growth-related traits of large yellow croaker *Pseudosciaena crocea* from Min-Yuedong tribe. Period. Ocean Univ. China. 38 (6):916-920. 10.16441/j.cnki.hdxb.2008.06.010

[B28] Liu Z. J. Cordes J. F. (2004). DNA marker technologies and their applications in aquaculture genetics. Aquac. 23 (1), 1–37. 10.1016/j.aquaculture.2004.05.027

[B29] Martínez P. Carmen B. Miguel H. Jesús F. Miguel Angel T. Manuel V. (2009). Identification of the major sex-determining region of turbot (*Scophthalmus maximus*). Genetics 183 (4), 1443. 10.1534/genetics.109.107979 19786621 PMC2787431

[B30] Neto R. V. R. Freitas R.T.F.d. Serafini M. A. (2012). Interrelationships between morphometric variables and rounded fish body yields evaluated by path analysis. Rev. Bras. Zootec. 41, 7. 10.1590/S1516-35982012000700004

[B31] Nichols K. M. Young W PDanzmann R. G. Robison B. D. Rexroad C. Noakes M. (2015). A consolidated linkage map for rainbow trout (*Oncorhynchus mykiss*). Anim. Genet. 34 (2), 102–115. 10.1046/j.1365-2052.2003.00957.x 12648093

[B32] Niu D. Du Y. Wang Z. Xie S. Nguyen H. Dong Z. (2017). Construction of the first high-density genetic linkage map and analysis of quantitative trait loci for growth-related traits in *Sinonovacula constricta*. Mar. Biotechnol. 19 (5): 488-496. 10.1007/s10126-017-9768-2 28725940

[B33] Palaiokostas C. Bekaert M. Davie A. Cowan M. E. Oral M. Taggart J. B. (2013). Mapping the sex determination locus in the Atlantic halibut (*Hippoglossus hippoglossus*) using RAD sequencing. BMC Genomics 14 (1), 566. 10.1186/1471-2164-14-566 23957753 PMC3765698

[B34] Palaiokostas C. Bekaert M. Taggart J.B. Gharbi K. Mcandrew B.J. Chatain B. (2015). A new SNP-based vision of the genetics of sex determination in European sea bass *(Dicentrarchus labrax)*. Genet. Sel. Evol. 47 (1), 68. 26337592 10.1186/s12711-015-0148-y PMC4558911

[B35] Palaiokostas C. Kocour M. Prchal M. Houston R. D. (2018). Accuracy of genomic evaluations of juvenile growth rate in common carp (*Cyprinus carpio*) using genotyping by sequencing. Front. Genet. 9, 82–. 10.3389/fgene.2018.00082 29593780 PMC5859378

[B36] Rastas P. Calboli F. C. F. Guo B. Shikano T. Merilä J. (2016). Construction of ultradense linkage maps with Lep-MAP2: Stickleback F 2 recombinant crosses as an example. Genome Biol. Evol. 8 (1), 78–93. 10.1093/gbe/evv250 PMC4758246 26668116

[B37] Reid D. P. Szanto A. Glebe B. Danzmann R. G. Ferguson M. M. (2005). QTL for body weight and condition factor in Atlantic salmon (*Salmo salar*): comparative analysis with rainbow trout (*Oncorhynchus mykiss*) and Arctic charr (*Salvelinus alpinus*). Heredity 94 (2), 166–172. 10.1038/sj.hdy.6800590 15483654

[B38] Rochette N. C. Catchen J. M. (2017). Deriving genotypes from RAD-seq short-read data using Stacks. Nat. Protoc. 12 (12), 2640. 10.1038/nprot.2017.123 29189774

[B39] Salem M. Al-Tobasei R. Ali A. (2018). Genome-wide association analysis with a 50K transcribed gene SNP-chip identifies QTL affecting muscle yield in rainbow trout. 10.1101/355792 PMC6157414 30283492

[B40] Yunjing S. Cuiyun L. U. Xiaofeng Z. Chao L. I. Lei C. Xiaowen S. (2014). Excavating the main effect of quantitative trait loci related to head length, head length and body length ratio in mirror carp (cyprinus carpio). J. Fish. China 38 (5), 625–631. 10.3724/SP.J.1231.2014.49089

[B41] Shao C. Niu Y. Rastas P. Liu Y. Xie Z. Li H. (2015). Genome-wide SNP identification for the construction of a high-resolution genetic map of Japanese flounder (*Paralichthys olivaceus*): applications to QTL mapping of *Vibrio anguillarum* disease resistance and comparative genomic analysis. DNA Research 22 (2), 161–170. 10.1093/dnares/dsv001 25762582 PMC4401326

[B42] Shen Z. G. Wang H. P. (2014). Molecular players involved in temperature-dependent sex determination and sex differentiation in teleost fish. Genet. Select. Evol. 46 (1), 26. 10.1186/1297-9686-46-26 PMC4108122 24735220

[B43] Shirak A. Eyal S. Avner C. Howe A. E. Raisa D. Noam Z. (2006). Amh and Dmrta2 genes map to tilapia (*Oreochromis* spp.) linkage group 23 within quantitative trait locus regions for sex determination. Genetics 174 (3), 1573–1581. 10.1534/genetics.106.059030 16951079 PMC1667067

[B44] Song W. Li Y. Zhao Y. Liu Y. Niu Y. Pang R. (2012). Construction of a high-density microsatellite genetic linkage map and mapping of sexual and growth-related traits in half-smooth tongue sole (*Cynoglossus semilaevis*). Plos One 7 (12), e52097. 10.1371/journal.pone.0052097 23284884 PMC3527371

[B45] Songlin C. Guojie Z. Changwei S. Quanfei H. Geng L. Pei Z. (2014). Whole-genome sequence of a flatfish provides insights into ZW sex chromosome evolution and adaptation to a benthic lifestyle. Nat. Genet. 46 (3), 253–260. 10.1038/ng.2890 24487278

[B46] Sun C. Niu Y. Ye X. Dong J. Hu W. Zeng Q. (2017). Construction of a high-density linkage map and mapping of sex determination and growth-related loci in the mandarin fish (*Siniperca chuatsi*). BMC Genomics 18 (1), 446. 10.1186/s12864-017-3830-3 28587594 PMC5461734

[B47] Sun C. F. Ye X. Tian Y. Y. Dong J. J. (2015). Simple sequence repeat-based analysis of the genetic diversity and population genetic structure of populations of *Siniperca chuatsi*. Genet. Mol. Res. GMR 14 (3), 9343. 10.4238/2015.August.10.15 26345868

[B48] Sundin K. Brown K. H. Drew R. E. Nichols K. M. Wheeler P. A. Thorgaard G. H. (2005). Genetic analysis of a development rate QTL in backcrosses of clonal rainbow trout, *Oncorhynchus mykiss*. Aqua. 247 (1), 75–83. 10.1016/j.aquaculture.2005.02.054

[B49] Takashi K. Wataru K. Satoshi T. Ayumi O. Takayoshi M. Naoki M. (2012). A trans-species missense SNP in Amhr2 is associated with sex determination in the tiger pufferfish, *Takifugu rubripes* (fugu). Plos Genet. 8 (7), e1002798. 10.1371/journal.pgen.1002798 22807687 PMC3395601

[B50] Uchino T. Nakamura Y. Sekino M. (2016). Constructing genetic linkage maps using the whole genome sequence of Pacific bluefin tuna (*Thunnus orientalis*) and a comparison of chromosome structure among teleost species. Adv. Biosci. Biotechnol. 7, 0. 10.4236/abb.2016.72010

[B51] Wang C. M. Lo L. C. Zhu Z. Y. Yue G. H. (2006). A genome scan for quantitative trait loci affecting growth-related traits in an F1 family of Asian seabass (*Lates calcarifer*). BMC Genomics 7 (1), 274–274. 10.1186/1471-2164-7-274 17064422 PMC1634999

[B52] Wang C. M. Zhi Y. B. Xiao P. H. Lin G. Xia J. H. Fei S. (2011). A high-resolution linkage map for comparative genome analysis and QTL fine mapping in Asian seabass, *Lates calcarifer*. BMC Genomics 12 (1), 174. 10.1186/1471-2164-12-174 21457569 PMC3088568

[B53] Wang W. Hu Y. Ma Y. Xu L. Guan J. Kong J. (2015). High-density genetic linkage mapping in turbot (*Scophthalmus maximus* L.) based on SNP markers and major sex- and growth-related regions detection. Plos One 10 (3), e0120410. 10.1371/journal.pone.0120410 25775256 PMC4361591

[B54] Xiaofeng Z. Yan Z. Xianhu Z. Youyi K. Zixia Z. Lan Z. (2013). A consensus linkage map provides insights on genome character and evolution in common carp (*Cyprinus carpio* L.). Mar. Biotechnol. 15 (3), 275–312. 10.1007/s10126-012-9485-9 23073608

[B55] Yu H. You X. Li J. Liu H. Meng Z. Xiao L. (2016). Genome-wide mapping of growth-related quantitative trait loci in orange-spotted grouper (*Epinephelus coioides*) using double digest restriction-site associated DNA sequencing (ddRADseq). Int. J. Mol. Sci. 17 (4), 501–. 10.3390/ijms17040501 27058532 PMC4848957

[B56] Yue G. H. (2014). Recent advances of genome mapping and marker-assisted selection in aquaculture. Fish Fish. 15 (3), 376–396. 10.1111/faf.12020

[B57] Yun L. Shikai L. Zhenkui Q. Geoff W. Ruijia W. Luyang S. (2015). Construction of a high-density, high-resolution genetic map and its integration with BAC-based physical map in channel catfish. DNA Research 22 (1), 39–52. 10.1093/dnares/dsu038 25428894 PMC4379976

[B58] Zhang G. Zhang X. Ye H. Jiang S. Hui Y. Jia L. (2017). Construction of high-density genetic linkage maps and QTL mapping in the golden pompano. Aquaculture 482, S0044848616311139. 10.1016/j.aquaculture.2017.09.011

[B59] Zhang N. Zhang L. Tao Y. Guo L. Sun J. Li X. (2015). Construction of a high density SNP linkage map of kelp (*Saccharina japonica*) by sequencing Taq I site associated DNA and mapping of a sex determining locus. BMC Genomics 16 (1), 1–11. 10.1186/s12864-015-1371-1 25887315 PMC4369078

[B60] Zhang Y. Xu P. Lu C. Kuang Y. Zhang X. Cao D. (2011). Genetic linkage mapping and analysis of muscle fiber-related QTLs in common carp (*Cyprinus carpio* L.). Mar. Biotechnol. 13 (3), 376–392. 10.1007/s10126-010-9307-x 20886255

[B61] Zhao Y. Peng W. Guo H. Chen B. Zhou Z. Xu J. (2017). Population genomics reveals genetic divergence and adaptive differentiation of Chinese sea bass (*Lateolabrax maculatus*). Mar. Biotechnol. 20 (Suppl), 45–59. 10.1007/s10126-017-9786-0 29256104

